# Interventional Treatments of Liver Metastases from Colorectal Cancer: Prognostic Factors and Subgroup Analyses

**DOI:** 10.3390/jcm14197027

**Published:** 2025-10-04

**Authors:** Thomas J. Vogl, Lars Hammann, Hannah Stefan, Leon Vincent Stein, Hamzah Adwan

**Affiliations:** Clinic for Radiology and Nuclear Medicine, University Hospital Frankfurt, Goethe University, Theodor-Stern-Kai 7, 60590 Frankfurt Am Main, Germany

**Keywords:** interventional radiology, transarterial chemoembolization, microwave ablation, liver metastases from colorectal cancer, overall survival, hepatic progression-free survival

## Abstract

**Background/Objectives**: This secondary analysis aims to evaluate various prognostic factors for overall survival (OS) and hepatic progression-free survival (hPFS) and to perform subgroup analyses regarding OS of patients with unresectable and chemotherapy-refractory liver metastases from colorectal cancer (LMCRC) treated by the combination therapy of transarterial chemoembolization (TACE) and microwave ablation (MWA) or with MWA alone. **Methods**: A total of 251 patients with unresectable and chemotherapy-refractory LMCRC were included retrospectively. Group A consisted of 184 patients who received a combination of TACE and MWA. A total of 67 patients were included in group B, who received only MWA. Group C summarizes the total number of 251 patients treated. For all groups, the influence of age, sex, number of metastases, the diameter and volume of the largest metastasis, and the occurrence of recurrence on OS and hPFS was determined using univariate cox regression analysis. OS was compared between patients with more than three metastases and those with three or fewer, as well as between patients with a diameter of largest metastasis of 3 cm or less with patients with a diameter of largest metastasis of more than 3 cm. The analysis of OS was carried out using the Kaplan–Meier method. **Results**: For all three groups, the parameters of age, sex, number of metastases, diameter and volume of the largest metastasis, complete ablation status, and recurrence were not significant prognostic factors for OS. The number of metastases had a statistically significant influence on hPFS in group C (*p* = 0.034) and trended towards significance in group A (*p* = 0.057). The mean OS of patients with three metastases or less was in all groups longer than patients with more than three metastases, however, with no statistically significant differences (*p* = 0.83, 0.451 and 0.84 for groups A, B and C, respectively). There were no significant differences regarding OS between patients with diameter of largest metastasis of 3 cm or less compared to patients with diameter of largest metastasis more than 3 cm in all groups (*p* = 0.316, 0.812 and 0.45 for groups A, B and C, respectively). **Conclusions**: We could not observe significant prognostic factors in the treatment of LMCRC on OS. Accordingly, patients with three metastases or less had non-significant longer OS than patients with more than three metastases. The number of metastases had a significant impact on hPFS of the whole patient cohort and borderline significant impact on hPFS in patients treated with TACE combined with MWA.

## 1. Introduction

The development of metastases represents a major challenge in the treatment of colorectal cancer [1,2]. Liver metastases from colorectal cancer (LMCRC), in particular, are predominant during the course of disease and significantly influence mortality [1,3].

In our publication from October 2024, we were already able to demonstrate a positive impact on hepatic distant tumor progression (hDTP), hepatic progression-free survival (hPFS), and overall survival (OS) in the treatment of LMCRC using a combination therapy composed of transarterial chemoembolization (TACE) and microwave ablation (MWA), compared to MWA monotherapy [4].

An important question that now warrants further investigation is the presence of potential prognostic factors within the observed cohort on OS and hPFS. Previous studies have already examined prognostic factors in the context of neoadjuvant treatment approaches. In these studies, a prolonged interval until the occurrence of liver metastases or the use of targeted agents in combination with chemotherapeutic regimens have been identified as factors significantly associated with prolonged OS [5].

Various studies have also identified potential predictors of OS in the context of surgical treatment approaches; however, the issue remains controversial [6]. Rectal localization of the primary tumor and the presence of more than three liver metastases have been associated with poorer OS [7]. Furthermore, levels above 5 ng/mL of carcinoembryonic antigen (CEA)—a blood-based tumor marker for colorectal carcinoma—are related to poorer OS along with R1 resection, where the tumor tissue extends beyond the resection margins in the histological section [7]. The presence of extrahepatic disease and a size above five centimeters in diameter are also seen as negative prognostic factors on OS in context of hepatectomy [1]. Beppu et al. [8] demonstrated that, in initially unresectable cases, conversion hepatectomy as well as response to chemotherapy and targeted therapy are independent prognostic factors for OS.

Molecular markers such as rat sarcoma viral oncogene family (RAS), v-Raf murine sarcoma viral oncogene homolog B1 (BRAF), and microsatellite instability (MSI) are also considered potential prognostic factors. A 2024 meta-analysis concluded that BRAF mutations in LMCRC patients after surgical resection are associated with significantly higher mortality and relapse [9]. Another study by Dijkstra et al. (2021) [10] showed a negative impact of BRAF and MSI mutations on the OS of patients with LMCRC. In their study, RAS mutations only led to reduced OS after ablation, but not after resection [10]. These studies highlight the increasing importance of molecular markers in the prognostic assessment of patients with LMCRC.

Our previous study evaluated different treatment strategies for unresectable and chemotherapy-refractory LMCRC, including combination therapy with TACE and MWA, as well as MWA monotherapy, investigating their efficacy and safety [4]. The aim of this secondary analysis is to investigate potential prognostic factors for OS as well as hPFS and to perform subgroup analyses of OS in patients receiving these locoregional treatment regimens.

## 2. Materials and Methods

### 2.1. Study Design

This secondary analysis was approved by the University Hospital’s Ethics Committee, which is based on our previously published study (Approval code: 2021–528) in October 2024 [4].

We considered patients until September 2021 and retrospectively reviewed data from a total of 251 patients with unresectable and chemotherapy-refractory LMCRC treated either with TACE followed by MWA (Group A, n = 184; 104 men and 80 women; mean age 64 ± 11.4 years; 442 metastases) or with MWA alone (Group B, n = 67; 49 men and 18 women; mean age: 63.2 ± 11.8 years; 173 metastases). Group C comprised the entire cohort (n = 251; 153 men and 98 women; mean age: 63.7 ± 11.5 years; 615 metastases).

We included patients above the age of 18 years with no more than five unresectable and chemotherapy-refractory LMCRC, with recent available magnetic resonance imaging that were treated by MWA with or without previous TACE. The patients should also have a minimum of one follow-up using magnetic resonance imaging. We applied the following exclusion criteria: (1) Liver metastasis of primary tumors other than colorectal cancer, (2) no available follow-up images, and (3) LMCRC size of > 5 cm. The focus of this secondary analysis was on determining prognostic factors, including age, sex, number of metastases, diameter and volume of the largest metastasis, complete ablation status, and recurrence on OS and hPFS. Furthermore, the number of TACE sessions was examined for group A.

Additionally, OS was compared between patients with more than three metastases and those with three or fewer as well as between patients with diameter of largest metastasis of 3 cm or less with patients with diameter of largest metastasis of more than 3 cm.

The characteristics of all three groups are summarized in Table 1.

### 2.2. Procedures

The protocols of TACE and MWA were carried out as thoroughly described and discussed in the initial publication [4]. TACE was performed by firstly establishing an arterial access usually through the femoral artery after local anesthesia. Chemotherapeutic agents such as Mitomycin C, Cisplatin, and Irinotecan were applied after reaching the tumor supplying arteries. The embolization was carried out using lipiodol.

MWA was performed percutaneously under the guidance of computed tomography after administering medications for analgesia and sedation. Firstly, a planning computed tomography scan was carried out to choose the best pathway for the microwave antenna toward the LMCRC. After that, a small incision was made, and the antenna was carefully inserted. The ablation procedure was then started after confirming the correct position of the MWA antenna. The patients were discharged on the same day after TACE or MWA, provided no complications occurred.

### 2.3. Statistics

For the statistical analysis of prognostic factors, univariate Cox regression analyses were carried out. In addition, Kaplan–Meier analyses were used to calculate the OS of the subgroups. Log-rank test was performed to determine the significance between subgroups. We considered a *p*-value of 5% or less as statistically significant. We used the IBM SPSS Statistics Version 30.0 for our statistical analyses.

## 3. Results

### 3.1. Analyses Regarding Prognostic Factors for OS

In Group A, none of the examined parameters (age, sex, diameter and volume of the largest metastasis, number of metastases, complete ablation status, number of TACE sessions, or recurrence) showed a significant influence on OS (Table 2).

In Group B, none of the analyzed variables, including age, sex, number of metastases, diameter and volume of the largest metastasis, complete ablation status, or recurrence, showed a significant effect on OS (Table 3).

In Group C, none of the examined parameter (age, sex, diameter and volume of the largest metastasis, number of metastases, complete ablation status, recurrence) had significant influence on OS (Table 4).

### 3.2. Analyses Regarding Prognostic Factors for hPFS

In Group A, none of the investigated factors (age, sex, diameter and volume of the largest metastasis, ablation status, recurrence, or number of TACE sessions) significantly influenced hPFS (Table 5). The number of metastases trended towards significance on hPFS (*p* = 0.057).

In Group B, hPFS was not significantly affected by age, sex, diameter and volume of the largest metastasis, number of metastases, complete ablation status, or recurrence (Table 6).

In Group C, the number of metastases was a significant factor for hPFS (*p* = 0.034), while age, sex, diameter and volume of the largest metastasis, complete ablation status, and recurrence were not significantly associated (Table 7).

### 3.3. Subgroup Analyses Regarding OS

Patients with three metastases or fewer had a longer mean OS than those with more than three in all groups, but without statistical significance (*p* = 0.83, 0.451, and 0.84 for groups A, B, and C, respectively).

Among patients with >3 metastases, the mean OS was slightly higher with TACE + MWA compared to MWA alone (40.5 vs. 37.5 months; *p* = 0.49). The 1-year OS was 81.1% of patients with more than three LMCRC treated by TACE + MWA, compared to 72.2% for patients with more than three LMCRC treated by MWA monotherapy.

We did not observe significant differences regarding OS between patients with diameter of largest metastasis of 3 cm or less compared to patients with diameter of largest metastasis more than 3 cm in all groups (*p* = 0.316, 0.812 and 0.45 for groups A, B and C, respectively).

## 4. Discussion

The aim of this retrospective study was to identify prognostic factors for OS and hPFS in patients with unresectable and chemotherapy-refractory LMCRC treated with locoregional treatments, including TACE and MWA as combination therapy and MWA as monotherapy. We also performed subgroup analyses regarding OS.

The included patients were divided into groups, depending on which therapy they received. A third group comprised all included patients.

The statistical analysis included parameters such as age, gender, number of metastases, diameter and volume of the largest metastasis, complete ablation status, or the occurrence of recurrence. None of the observed factors could be demonstrated to have a significant impact on OS. The mean OS of patients with more than three metastases compared to patients with less than or equal to three metastases was also not significantly lower in all three groups. No significant differences were observed regarding OS of patients with diameter of largest metastasis of 3 cm or less compared to patients with diameter of largest metastasis more than 3 cm in all groups. In the analysis of prognostic factors on hPFS, the number of initial metastases in Group C could be seen as a statistically significant factor (*p* = 0.034) and trending towards significance in Group A (*p* = 0.057). The missing significant influence of initial metastases on OS, despite an association with hPFS, may be explained by the multifactorial nature of OS, whereas hPFS more directly reflects the efficacy of local liver-directed therapy. This aspect is likely to account for the discrepancy between the two endpoints.

A study by Liang et al. [11] was published in 2003. Seventy-four patients were treated with percutaneous microwave coagulation therapy, and the focus was on survival and prognostic factors. As in our study, age (*p* = 0.46) and gender (*p* = 0.12) had no influence on survival rates. The site of primary malignancies also had no significant effect on OS (*p* = 0.58) in a univariate analysis. However, Liang et al. [11] were able to demonstrate a significance on survival of the following factors in their multivariate analysis: number of metastases (*p* = 0.03) and local recurrence or new metastasis (*p* = 0.04). The size of tumors showed no significant influence on OS in the multivariate analysis (*p* = 0.408) [11].

Another study also investigated prognostic factors for OS in patients with LMCRC treated with MWA. In 2014, Wang et al. [12] published this study and found that there was no statistical influence of gender (*p* = 0.155), age (*p* = 0.806), number of metastases (*p* = 0.571), and metastasis size (*p* = 0.086) on OS. The results of this study, therefore, correspond well with our study results.

In a systematic review by Klubien et al. (2018) [13], 18 studies investigating colorectal liver metastases treated with MWA, radiofrequency ablation, or laser ablation were analyzed. Regarding OS, the review also found no impact of the number of metastases. Only three of the included studies investigated the influence of tumor size on OS, with most reporting no association. Prognostic factors related to local tumor control, such as local tumor progression, also showed no statistically significant correlation with tumor size or number of metastases. Our findings are, therefore, consistent with the current literature regarding OS. However, regarding hPFS, a statistically significant influence of the number of metastases could be observed in our study.

The investigation of molecular markers could significantly expand the identification of prognostic risk factors. A comparable retrospective study found that patients with RAS mutations in LMCRC had a shortened PFS after thermal ablation [14]. Another study describes the association of RAS mutation with a worse prognosis after thermal ablation, but not after resection [10]. A consequence of identifying RAS as a prognostic risk factor in thermal ablation could, therefore, be a need for larger ablation zones when this molecular mutation is present [10]. Future studies on risk factors with a focus on molecular mutations could further help to improve therapy recommendations for specific patient subgroups.

This study has limitations that should be mentioned. Despite the high number of included patients, this study is mainly limited due to the retrospective design and potential selection bias, among others. The mutation status of colorectal carcinomas, such as BRAF, MSI, or RAS was not assessed in this study. It is important to highlight that the mutation status of colorectal cancer may have significant impact on the outcomes and survival of the patients. Furthermore, there was no exact consideration of the indications for the treatments. A randomized, prospective study could contribute to a better comparison of subgroup analysis and the identification of prognostic factors.

In summary of the existing study situation, it can be stated that there is still disagreement regarding prognostic factors for OS in patients with LMCRC that have been treated with locoregional therapy methods. Further prospective multicenter studies should, therefore, be carried out to expand the data situation.

## 5. Conclusions

We conclude that we did not observe any significant prognostic factors for OS in patients with unresectable and chemotherapy-refractory LMCRC treated by therapy protocols using TACE and MWA in combination, as well as MWA alone. The number of metastases had significant impact on hPFS of the whole patient cohort and trending towards significant impact on hPFS of the patients treated by the combination therapy. Patients with three metastases or less had non-significant longer OS than patients with more than three metastases. Also, the OS of patients with a diameter of largest metastasis of 3 cm or less did not significantly differ from the OS of patients with a diameter of largest metastasis more than 3 cm.

## Figures and Tables

**Table 1:** Characteristics of patient groups.

Parameter	Group A	Group B	Group C
Number of patients	184	67	251
Women n (%)	80 (43.5)	18 (26.9)	98 (39)
Men n (%)	104 (56.5)	49 (73.1)	153 (61)
Mean age	64 ± 11.4 years	63.2 ± 11.8 years	63.7 ± 11.5 years
Total number of metastases	442	173	615
Patient with one metastases n (%)	49 (26.6)	20 (29.9)	69 (27.5)
Patients with two metastases n (%)	67 (36.4)	22 (32.8%)	89 (35.5)
Patients with three metastases or more n (%)	68 (37)	25 (37.3)	93 (37)
Number of metastases less than 2 cm n (%)	219 (49.5)	91 (52.8)	310 (50.4)
Diameter of metastases ≥ 2–3 cm n (%)	139 (31.5)	59 (34.1)	198 (32.2)
Number of metastases equal to 3 cm or larger n (%)	84 (19)	23 (13.1)	107 (17.4)

**Table 2:** Prognostic factors for OS (Group A).

Prognostic Factors	*p*-Value	Hazard Ratio	95% Confidence Interval: Lower	95% Confidence Interval: Upper
Age	0.856	0.998	0.981	1.016
Sex	0.265	1.265	0.837	1.913
Number of metastases	0.717	1.026	0.893	1.179
Recurrence	0.984	0.995	0.617	1.604
Diameter of the largest metastasis	0.09	0.988	0.974	1.002
Volume of the largest metastasis	0.091	0.989	0.977	1.002
Complete ablation status	0.348	0.672	0.293	1.541
Number of TACE before MWA treatment	0.137	1.051	0.984	1.123

**Table 3:** Prognostic factors for OS (Group B).

Prognostic Factors	*p*-Value	Hazard Ratio	95% Confidence Interval: Lower	95% Confidence Interval: Upper
Age	0.292	0.983	0.951	1.015
Sex	0.917	1.037	0.520	2.067
Number of metastases	0.618	1.039	0.894	1.207
Recurrence	0.126	0.498	0.204	1.217
Diameter of the largest metastasis	0.819	0.998	0.979	1.017
Volume of the largest metastasis	0.564	1.005	0.989	1.021
Complete ablation status	0.228	0.415	0.099	1.736

**Table 4:** Prognostic factors for OS (Group C).

Prognostic Factors	*p*-Value	Hazard Ratio	95% Confidence Interval: Lower	95% Confidence Interval: Upper
Age	0.468	0.994	0.979	1.010
Sex	0.28	1.215	0.853	1.729
Number of metastases	0.629	1.025	0.927	1.133
Recurrence	0.524	0.873	0.574	1.327
Diameter of the largest metastasis	0.126	0.991	0.980	1.002
Volume of the largest metastasis	0.141	0.993	0.984	1.002
Complete ablation status	0.159	0.597	0.292	1.224

**Table 5:** Prognostic factors for hPFS (Group A).

Prognostic Factors	*p*-Value	Hazard Ratio	95% Confidence Interval: Lower	95% Confidence Interval: Upper
Age	0.709	0.997	0.983	1.011
Sex	0.908	0.981	0.712	1.352
Number of metastases	0.057	1.128	0.996	1.277
Diameter of the largest metastasis	0.904	0.999	0.990	1.009
Volume of the largest metastasis	0.793	1.001	0.995	1.006
Complete ablation status	0.836	1.065	0.588	1.930
Number of TACE before MWA treatment	0.185	1.039	0.982	1.099

**Table 6:** Prognostic factors for hPFS (Group B).

Prognostic Factors	*p*-Value	Hazard Ratio	95% Confidence Interval: Lower	95% Confidence Interval: Upper
Age	0.723	1.004	0.982	1.027
Sex	0.461	0.807	0.456	1.428
Number of metastases	0.404	1.061	0.923	1.221
Diameter of the largest metastasis	0.364	1.007	0.992	1.023
Volume of the largest metastasis	0.178	1.009	0.996	1.022
Complete ablation status	0.304	1.564	0.667	3.666

**Table 7:** Prognostic factors for hPFS (Group C).

Prognostic Factors	*p*-Value	Hazard Ratio	95% Confidence Interval: Lower	95% Confidence Interval: Upper
Age	0.824	0.999	0.987	1.011
Sex	0.905	0.983	0.746	1.296
Number of metastases	0.034	1.103	1.008	1.207
Diameter of the largest metastasis	0.908	1.000	0.992	1.009
Volume of the largest metastasis	0.635	1.001	0.996	1.006
Complete ablation status	0.464	1.199	0.738	1.950

## Data Availability

In justified cases, the data sets can be requested. It should be noted that all analyzed data has already been published in this paper.

## Abbreviations

The following abbreviations are used in this manuscript:

OS	Overall Survival
hPFS	Hepatic progression free survival
LMCRC	Liver metastases from colorectal cancer
TACE	Transaterial chemoembolization
MWA	Microwave ablation
hDTP	Hepatic distant tumor progression
CEA	Carcinoembryonic antigen
BRAF	v-Raf murine sarcoma viral oncogene homolog B1
MSI	Microsatellite instability
RAS	Rat sarcoma viral oncogene family
